# Utilization of alternative systems of medicine as health care services in India: Evidence on AYUSH care from NSS 2014

**DOI:** 10.1371/journal.pone.0176916

**Published:** 2017-05-04

**Authors:** Shalini Rudra, Aakshi Kalra, Abhishek Kumar, William Joe

**Affiliations:** 1 Associate Fellow, Observer Research Foundation, New Delhi, India; 2 Population Research Centre, Institute of Economic Growth, Delhi University North Campus, Delhi, India; Universita degli Studi di Firenze, ITALY

## Abstract

AYUSH, an acronym for Ayurveda, Yoga and Naturopathy, Unani, Siddha, Sowa-Rigpa and Homeopathy represents the alternative systems of medicine recognized by the Government of India. Understanding the patterns of utilization of AYUSH care has been important for various reasons including an increased focus on its mainstreaming and integration with biomedicine-based health care system. Based on a nationally representative health survey 2014, we present an analysis to understand utilization of AYUSH care across socioeconomic and demographic groups in India. Overall, 6.9% of all patients seeking outpatient care in the reference period of last two weeks have used AYUSH services without any significant differentials across rural and urban India. Importantly, public health facilities play a key role in provisioning of AYUSH care in rural areas with higher utilization in Chhattisgarh, Kerala and West Bengal. Use of AYUSH among middle-income households is lower when compared with poorer and richer households. We also find that low-income households display a greater tendency for AYUSH self-medication. AYUSH care utilization is higher among patients with chronic diseases and also for treating skin-related and musculo-skeletal ailments. Although the overall share of AYUSH prescription drugs in total medical expenditure is only about 6% but the average expenditure for drugs on AYUSH and allopathy did not differ hugely. The discussion compares our estimates and findings with other studies and also highlights major policy issues around mainstreaming of AYUSH care.

## Introduction

Traditional medicine is defined as an amalgamation of knowledge, skill, and practices based on theories, beliefs, and experiences indigenous to different cultures, whether explicable or not, used for therapeutic, restorative, prevention, diagnosis and maintenance of physical and mental health [1,2]. These systems are recognised globally for complementing disease prevention, treatment and generic health maintenance [1,3,4]. There is widespread use of traditional medicine across developing countries (across Asia, Africa and Latin America) with rapidly emerging markets in North America and Europe [2,3]. However, despite increasing national and international attention, the formal health systems, particularly in resource-poor settings, are yet to harness its true potential. Recognising such intricacies, the 67^th^ World Health Assembly resolution on traditional medicine has been instrumental in the development of updated WHO Traditional Medicine Strategy (2014–23) with objectives to harness its contribution and promote effective use [2].

These international developments are of particular significance for India that has a pluralistic medical culture with a well-documented history and practice of alternative medicinal forms namely—Ayurveda, Yoga and Naturopathy, Unani, Siddha and Homoeopathy–now jointly referred to as AYUSH [5–10]. As integral part of Indian culture, Indian System of Medicine (ISM) comprising of Ayurveda, Yoga, Unani and Siddha was practiced even before formal health system took shape. Unfortunately, in spite of presenting an effective role for health maintenance (preventive and curative), the ISM has been neglected and marginalized since the pre-independence era (before 1947). Prejudiced approach like curbing the state patronage, abolition of ISM schools and creation of medical bureaucracy further undermined the legitimacy of ISM practice [11,12]. The potential of ISM was further curbed by the perils of caste-, class-, communal-and language-based politics. Besides, strong epistemological preferences within ISM system undermined its scope and expansion as a formal health care system in the post-independence era. [13–18]. However, time and again, with the realization of strengthening comprehensive primary health care, various Expert Groups and Health Committees in India have recommended integration of ISM with the biomedical/allopathic system. For instance, Mudaliar Committee was first to recommend utilizing indigenous doctors for delivering vertical healthcare programs [19]. Subsequent national health policies such as National Health Policy (1983), National Education Policy in Health Sciences in 1989 and National Health Policy (2002) have also pointed out the potential of ISM in improving healthcare access, particularly in the absence of modern healthcare in rural India. But attempts made towards its revival thereafter, only saw bio-medicalization of these systems either for validation through scientific methods or for commercialization and excessive marketization of indigenous therapies. However, the paradigm shift in government’s policy post-independence has focused on: creation of schools for ISM to produce licentiates, standardizing curriculum, drug research for patenting and mass production. The institutionalization of ISM in keeping with international interests further led to the creation of the Department of Indian Systems of Medicine and Homeopathy (ISM&H) in 1995 (later renamed as Department of AYUSH in 2003). In pursuance of ‘mainstreaming’ policy, post-2005, National Rural Health Mission (NRHM) provided an opportunity where strategies like provisioning of AYUSH drugs, co-locating providers at public health facilities and inter-sectoral convergence with ISM functionaries implementing national health programs were devised [20]. Department of AYUSH also launched National AYUSH Mission during 12^th^ plan with an objective of providing affordable, sustainable and accessible care. Subsequently, the elevation of AYUSH Department into an independent Ministry (in November 2014) is a noteworthy policy decision to further upgrade AYUSH educational standards with emphasis on epistemological strengths, quality standardization and stewardship.

Policies notwithstanding, existing research on AYUSH medicine in India is too sporadic and dispersed to facilitate an understanding of AYUSH care utilization [5,8,9, 21–29]. It may be noted that much of the earlier evidence on use of traditional medical services comes from small area studies [24–27] and there are only a few studies based on a sample large enough to generate any evidence [5,29,30]. Clearly, there is a need to undertake more systematic analysis to examine AYUSH care utilization across regional, socioeconomic and demographic groups [5]. Also, in a country like India which is home to many traditional medicine systems, it is also essential to understand these patterns in conjunction with allopathic medicine. This paper therefore aims to address some of these gaps by contributing to the evidence base regarding utilization of AYUSH health care services in India. The analysis is based on a nationally representative cross-sectional household survey conducted in 2014 by National Sample Survey Organization (NSSO), Government of India [31]. It is expected that the findings will provide vital insights regarding utilization patterns as well as socioeconomic profile of users and contribute towards the discussions on integrating AYUSH within the formal healthcare system in India.

## Data and methods

The analysis is based on data from a nationally representative cross-sectional survey on Social Consumption: Health (Schedule 25.0, 71^st^ round, January–June 2014) which covered whole of the Indian Union (for details of the survey and interview schedule see http://mail.mospi.gov.in/index.php/catalog/161/related_materials). A stratified multi-stage sampling strategy was employed for this survey with a total sample size of 65,932 households (36,480—rural and 29,452—urban) comprising of 333,104 individuals (189,573—rural and 143,531—urban). The survey captures information regarding health and health care utilization and elicits information regarding nature of treatment received by patients for outpatient and inpatient care. The classification codes for nature of treatment provided by NSSO included Allopathy, ISM (includes Ayurveda, Siddha, Unani and Sowa-Rig-Pa), Homeopathy and Yoga & Naturopathy (see supplemental file). It is important to note that home-based remedies and folk medicine were included under the ambit of ISM only. The above mentioned classification codes for ISM, Homeopathy and Yoga & Naturopathy were combined and referred to as AYUSH in the survey, representing the use of any of the traditional systems of medicine by the patients. It may be noted that the survey does not provide any information to distinguish whether the providers of AYUSH care do or do not hold desirable AYUSH qualifications. For analytical purposes, the study uses information related to use of AYUSH services for outpatient care with a recall period of last 15 days. We did not include analysis of inpatient care because the use of AYUSH for hospital-based care is very low (below 1% of all hospitalization cases). The survey also provides information regarding socioeconomic background of the surveyed households (see S1 Table for descriptive statistics). Here, the monthly per-capita household consumer expenditure (MPCE) information is used as a proxy for income and separate MPCE quintiles are constructed for rural and urban areas to depict their relative economic status.

We describe utilization of different forms of medicine, across type of ailment and related out of pocket expenditure across rural and urban India. We also report the association between economic status and utilization of AYUSH services using concentration curve (CC) and concentration index (CI) [32,33]. The CC plots the cumulative proportions of treatment-seeking patients ranked by MPCE (beginning with the most disadvantaged in terms of MPCE and ending with the least disadvantaged) on the x-axis against the cumulative proportions of patients using different forms of treatment–allopathy, ISM or AYUSH on y-axis. If use of a particular nature of treatment is equally distributed across MPCE, the CC will coincide with the diagonal (line of equality) and if it is concentrated among higher MPCE classes, then CC lies below the diagonal. For interpretative purposes, further the CC from the diagonal, the greater would be the degree of inequality. The CI could be derived from the CC and is defined as twice the area between the CC and the diagonal. The CI can be written in many ways, one being [33];

$$\text{C}\left(\text{h}\right)=\frac{2}{n^{2}\mu_{h}}\overset{n}{\underset{i=1}{\sum }}z_{i}h_{i}$$

Where $z_{i}=\frac{n+1}{2}-\lambda_{i}$

where, i:(i = 1, 2,…, n) represents a given population; λ_i_ s the MPCE rank of the person with the best well-off individual ranked first and the least well-off ranked last. In the case of ties, each member of the tied group is assigned the average rank of the group. The CI ranges between +1 and -1 with zero depicting no inequality and large positive values suggesting higher use of a particular nature of treatment among the richer sections.

The econometric analysis further explores the socioeconomic correlates of AYUSH use. It recognizes that health care utilization involves at least two steps: first, to decide whether to seek any health care when ill and second, to decide about the nature of care (AYUSH or others). Besides, the decision to seek health care is not only contingent upon experience of illness but also depends on various social, economic and demographic factors. Hence, if this decision is influenced by unobservable background characteristics then it is important to adjust for such selection bias before drawing any inferences regarding association of various socioeconomic correlates with AYUSH use. For this purpose, we employ Heckman probit regression for the econometric analysis [34–36] that captures the two-stage decision-making process–first decision to use health care (selection part) and second to use AYUSH care (treatment part)–and presents estimates adjusted for selection bias. We also use this approach to examine the decision to utilize AYUSH based on medical advice because it is likely that some patients may resort to self-medication practices. It may be noted that estimation of the selection model may require exclusion restrictions such that these variables are expected to influence the selection equation but should have no direct influence on the treatment equation. In our analysis, we identified reporting of illness (other than chronic illness) in the last 15 days as instrument that directly affect health care utilization but is less likely to influence choice regarding AYUSH care. Similarly, in the second model we used reporting of chronic illness as instrument that directly affects use of AYUSH care but is less likely to influence decision regarding use subject to medical advice. We also report the likelihood ratio test statistics for validity of the exclusion restrictions. All analyses were carried out using Stata version 12. This study is based on analysis of anonymized secondary data in the public domain and can be obtained from the NSSO.

## Results

Tables 1 and 2 report the distribution of ailing persons (patients) receiving outpatient care in the last 15 days by nature of treatment and background characteristics in rural and urban India, respectively. The distribution of patients presented under different nature of treatment is not necessarily mutually exclusive. This is because some patients may have experienced more than one spell of illness during the reference period and may have used different nature of treatment for different spells of ailment. The results presented in Tables 1 and 2 show that allopathic system has a dominant presence across rural and urban areas with over 90% of treatment-seeking patients across all socioeconomic groups reporting receipt of allopathy care (S2 and S3 Tables presents estimates adjusted for State as random effects). In rural and urban India, 93.4% and 93.5% patients (persons reporting illness) respectively, have received allopathy-based outpatient care in the last 15 days prior to the survey whereas during the same reference period AYUSH care was used by about 6.7% and 7.1% patients in rural and urban India, respectively. The ISM is the key component of the AYUSH system with utilization over 3.4% rural and 3.7% urban patients. Homeopathy also has a significant presence within the AYUSH system. Across age groups, use of ISM care is relatively high among elderly patients (4.1% and 4.8% in rural and urban India, respectively) while homeopathy care is relatively more among children (under-five years), particularly in urban areas (4.8%). Compared to males, use of AYUSH care among females was relatively high in rural India whereas no such gender-differential was observed in urban areas. Notably, use of AYUSH care was less observed among the middle MPCE quintile households whereas it was higher among those at the either end of the MPCE distribution (see S4 and S5 Tables). Also, patients with chronic illness reported greater use of AYUSH care both across rural (8.8%) and urban India (8.1%). While the all-India figures (Table 3) suggest that the AYUSH care was utilized by 6.9% of patients but there were considerable variations across states and union territories with nine of them having more than 10% patients utilizing AYUSH care (see S6 Table). Among major States, Chhattisgarh (15.4%), Kerala (13.7%), and West Bengal (11.6%) displayed the highest AYUSH utilization levels. While ISM system was more popular component of AYUSH in Chhattisgarh and Kerala, homeopathy had a dominant presence in West Bengal.

**Table 1:** Percentage patients (persons reporting illness during reference period of last 15 days) receiving medical treatment (excluding hospitalization) by nature of treatment and background characteristics, Rural India, 2014 (n = 13927).

Rural India	Allopathy	ISM	Homoeopathy	Yoga & Naturopathy	Other	AYUSH
**Age**						
Below 5 years	95.7	1.0	2.3	0.1	0.9	3.5
5 to 14 years	95.0	2.7	1.1	0.9	0.3	4.7
15 to 59 years	92.7	3.8	3.3	0.5	0.4	7.5
60 years and above	93.0	4.1	2.9	0.6	0.1	7.6
**Sex**						
Male	94.5	3.1	1.9	0.7	0.3	5.7
Female	92.5	3.7	3.7	0.4	0.5	7.6
**Social group**						
Scheduled Tribes	91.4	5.4	0.6	1.2	1.4	7.2
Scheduled Castes	94.6	2.6	2.2	0.7	0.4	5.5
Other Backward Classes	94.1	3.1	2.8	0.4	0.1	6.3
Others	92.0	3.8	4.1	0.4	0.5	8.1
**Religion**						
Hinduism	93.6	3.5	2.6	0.5	0.4	6.5
Islam	91.4	3.2	5.6	0.1	0.2	8.8
Others	94.8	3.0	1.2	1.3	0.5	5.5
**Education of Head**						
Illiterate	93.7	3.0	2.7	0.4	0.4	6.1
Primary or below	93.9	3.9	2.1	0.7	0.3	6.6
Secondary education	92.2	3.7	4.2	0.6	0.2	8.3
Higher education	92.5	3.5	3.7	0.0	1.3	7.2
**MPCE quintile**						
Lowest	92.6	3.2	2.7	1.0	0.4	6.9
Second	93.2	3.9	2.3	0.1	0.8	6.3
Middle	93.9	2.3	3.7	0.4	0.2	6.4
Fourth	95.1	3.1	1.9	0.2	0.2	5.2
Highest	92.3	4.1	3.5	0.7	0.4	8.2
**Chronic illness**						
Yes	91.9	4.0	4.2	0.7	0.4	8.8
**Acute illness**						
Yes	94.7	3.2	1.8	0.4	0.4	5.3
**Rural India**	93.4	3.4	2.8	0.5	0.4	6.7

Source: Authors using NSSO 71^st^ Round on Social Consumption: Health (2014)

It may be noted that some individuals may have received treatment from more than one forms of medicine and therefore the distribution of patients under nature of treatment is not mutually exclusive.

**Table 2:** Percentage patients (persons reporting illness during reference period of last 15 days) receiving medical treatment (excluding hospitalization) by nature of treatment and background characteristics, Urban India, 2014 (n = 13505).

Urban India	Allopathy	ISM	Homoeopathy	Yoga & Naturopathy	Other	AYUSH
**Age**						
Below 5 years	92.2	2.9	4.3	0.3	0.4	7.4
5 to 14 years	93.8	1.6	3.8	0.9	0.0	6.3
15 to 59 years	93.5	3.8	3.2	0.2	0.5	7.2
60 years and above	93.8	4.8	2.4	0.1	0.1	7.2
**Sex**						
Male	93.2	4.4	2.4	0.3	0.5	7.1
Female	93.8	3.2	3.7	0.2	0.2	7.1
**Social group**						
Scheduled Tribes	94.5	3.5	1.7	0.6	0.0	5.8
Scheduled Castes	94.5	4.1	2.0	0.2	0.2	6.3
Other Backward Classes	93.6	4.0	3.0	0.2	0.3	7.3
Others	93.0	3.3	3.7	0.3	0.5	7.3
**Religion**						
Hinduism	93.5	3.8	3.1	0.3	0.3	7.2
Islam	93.2	3.2	3.9	0.0	0.5	7.1
Others	94.2	4.1	2.2	0.1	0.1	6.4
**Education of Head**						
Illiterate	94.3	2.9	3.0	0.1	0.2	5.9
Primary or below	94.1	3.3	3.5	0.2	0.2	7.0
Secondary education	93.6	4.0	2.4	0.6	0.3	6.9
Higher education	91.6	5.1	3.9	0.0	0.8	9.0
**MPCE quintile**						
Lowest	94.9	2.3	2.9	0.1	0.1	5.3
Second	91.8	4.8	3.8	0.1	0.3	8.7
Middle	93.6	3.1	3.1	0.8	0.1	7.0
Fourth	94.9	3.3	1.8	0.1	0.7	5.2
Highest	92.7	4.6	4.0	0.1	0.4	8.7
**Chronic illness**						
Yes	93.1	4.4	3.6	0.2	0.5	8.1
**Acute illness**						
Yes	94.1	2.9	3.0	0.3	0.3	6.2
**Urban India**	93.5	3.7	3.2	0.2	0.3	7.1

Source: Authors using NSSO 71^st^ Round on Social Consumption: Health (2014)

It may be noted that some individuals may have received treatment from more than one forms of medicine and therefore the distribution of patients under nature of treatment is not mutually exclusive.

**Table 3:** Percentage patients (persons reporting illness during reference period of last 15 days) receiving medical treatment (excluding hospitalization) by nature of treatment and State/UT, 2014.

State	Allopathy	ISM	Homoeopathy	Yoga & Naturopathy	Other	AYUSH
A & N Islands	83.8	9.6	0.6	1.9	4.3	12.1
Andhra Pradesh	96.8	0.2	1.7	0.9	0.7	2.7
Arunachal Pradesh	81.6	13.9	0.8	0.0	3.7	14.7
Assam	97.1	1.5	1.2	0.0	0.2	2.7
Bihar	91.0	4.9	3.9	0.0	0.3	8.7
Chandigarh	91.4	8.2	0.4	0.0	0.0	8.6
Chhattisgarh	82.6	12.3	2.6	0.5	3.9	15.4
D & N Haveli	84.3	14.4	0.0	1.4	0.0	15.7
Daman & Diu	99.4	0.0	0.6	0.0	0.0	0.6
Delhi	99.7	0.1	0.0	0.2	0.0	0.3
Goa	99.2	0.5	0.1	0.0	0.1	0.7
Gujarat	97.2	3.7	0.0	0.7	0.0	4.3
Haryana	93.8	3.3	2.0	0.2	1.0	5.5
Himachal Pradesh	90.4	8.7	0.4	0.3	0.9	9.3
Jammu & Kashmir	98.8	0.3	0.8	0.2	0.0	1.2
Jharkhand	93.7	1.1	1.3	4.2	0.0	6.6
Karnataka	96.5	2.6	0.4	0.4	0.0	3.5
Kerala	88.9	8.8	4.8	0.1	0.8	13.7
Lakshadweep	88.8	7.9	3.3	0.0	0.0	11.2
Madhya Pradesh	96.0	3.3	0.7	0.0	0.1	4.0
Maharashtra	96.2	2.3	0.9	0.6	0.1	3.8
Manipur	99.7	0.0	0.0	0.0	0.3	0.0
Meghalaya	74.5	20.5	0.0	0.0	5.0	20.5
Mizoram	73.7	25.3	0.7	0.3	0.0	26.3
Nagaland	82.2	3.2	0.0	0.0	14.7	3.2
Odisha	91.8	4.2	3.3	0.1	0.7	7.5
Puducherry	97.4	1.6	1.0	0.0	0.0	2.6
Punjab	96.0	2.1	0.5	0.3	1.4	2.9
Rajasthan	93.6	5.7	0.9	0.1	0.0	6.7
Sikkim	99.9	0.1	0.0	0.0	0.0	0.1
Tamil Nadu	96.9	2.7	1.1	0.1	0.0	3.8
Telangana	98.5	1.0	0.1	0.3	0.0	1.5
Tripura	95.0	0.0	5.0	0.0	0.0	5.0
Uttar Pradesh	91.4	4.6	3.4	0.5	0.2	8.6
Uttarakhand	94.9	3.0	1.0	0.0	1.0	4.0
West Bengal	89.1	1.7	9.6	0.5	0.3	11.6
**All**	**93.4**	**3.5**	**3.0**	**0.4**	**0.4**	**6.9**

Source: Authors using NSSO 71^st^ Round on Social Consumption: Health (2014)

It may be noted that some individuals may have received treatment from more than one forms of medicine and therefore the distribution of patients under nature of treatment is not mutually exclusive.

**Fig 1:**
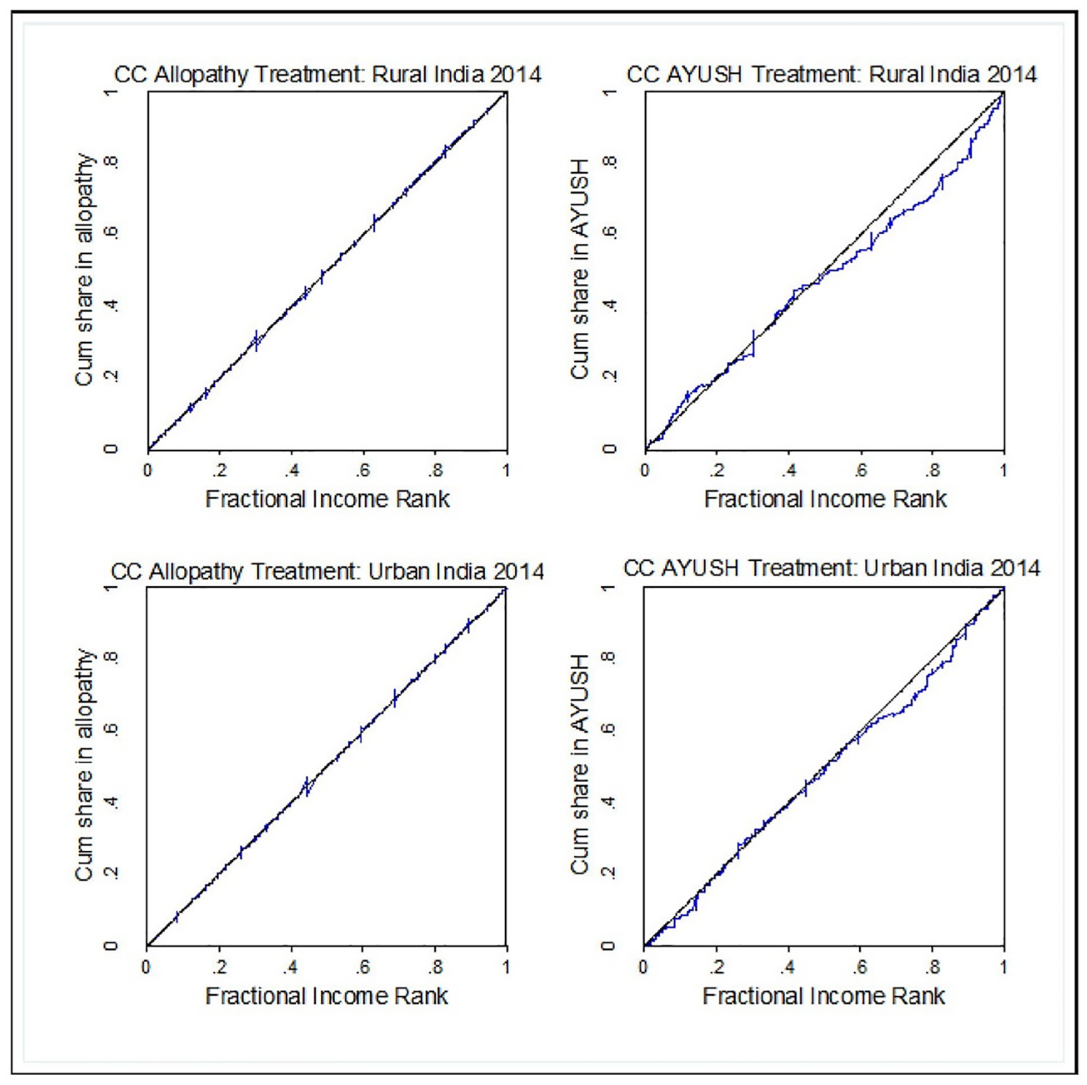
Concentration curves for nature of treatment–Allopathy and ISM—In last 15 days (excluding hospitalization), Rural and Urban India, 2014. Source: Authors using NSSO 71^st^ Round on Social Consumption: Health (2014).

Further, we use CC and CI to examine whether or not utilization of a particular nature of treatment for outpatient care was associated with socio-economic status (MPCE) of the ailing individuals. As shown in Fig 1, both in rural and urban areas, the CCs for allopathy care coincides with the diagonal indicating that allopathic care is almost equally used by patients from various MPCE levels. The CC for AYUSH care though coincides with the diagonal for the poorer sections but it deviates from the diagonal for the middle income and richer sections. The shape of the CC suggests there is differential in use of AYUSH care between middle MPCE and higher MPCE classes with relatively greater concentration observed among richer patients. However, whether or not the deviation from the diagonal is statistically significant needs to be confirmed based on the CI. Table 4 shows that the CI value for AYUSH use is positive and significant for the rural areas (CI: 0.047) but is statistically insignificant in urban areas. This implies that, unlike rural areas, there is no statistically significant difference in use of AYUSH across patients with varying MPCE class in urban areas. Furthermore, a zero value of the CI index for allopathy care validates this finding. Use of Homoeopathy, Yoga and Naturopathy does not display any consistent difference across MPCE classes. However, the positive and significant CI values for ISM (CI rural: 0.064 and CI urban: 0.074) does reveal a pro-rich tendency in utilization.

**Table 4:** Concentration indices for nature of treatment used in last 15 days (excluding hospitalization), Rural and Urban India, 2014.

Nature of treatment	CI for Rural India (se)		CI for Urban India (se)	
Allopathy	-0.001	*(0*.*001)*	-0.001	*(0*.*001)*
Indian System of Medicine	0.064*	*(0*.*026)*	0.074*	*(0*.*025)*
Homeopathy	0.049	*(0*.*028)*	-0.008	*(0*.*028)*
Yoga and Naturopathy	-0.006	*(0*.*068)*	-0.177	*(0*.*102)*
Other treatment	-0.062	*(0*.*078)*	0.239*	*(0*.*085)*
AYUSH	0.047*	*(0*.*018)*	0.029	*(0*.*018)*

Source: Authors using NSSO 71^st^ Round on Social Consumption: Health (2014)

Note: *Standard error* of the CI in parenthesis

* Denotes significance at 5% level.

We also explore the out of pocket expenditure on AYUSH treatment. Table 5 shows that, the average OOPE on AYUSH medicines is Rs 270 in rural and Rs 378 in urban areas and is lower than average expenditure on non-AYUSH (allopathic) medicine. The OOPE for males and females in urban areas is higher compared to their rural counterparts. We also find that the overall share of AYUSH medicines in total medical expenditure for outpatient care is 4.5% and 4.7% for rural and urban India, respectively. The total expenditure on AYUSH medicine accounted for about 6% of the total medicinal (drugs) expenditure for outpatient care. It is also worth noting that only 1.5% patients had received free AYUSH medicines during outpatient care whereas about 12% patients had received free allopathic medicines from public health facilities.

**Table 5:** Average out of pocket expenditure on AYUSH medicines and other (non-AYUSH) medicines per treated person in the last 15 days by sex and place of residence, 2014.

OOP expenditure (in Rupee)	Rural India (std. err)			Urban India (std. err)		
	Male	Female	All	Male	Female	All
AYUSH medicine	322	228	270	462	311	378
	(37.4)	(16.0)	(19.2)	(36.9)	(26.4)	(22.1)
Other (non-AYUSH) medicine	381	402	392	485	430	454
	(7.8)	(10.0)	(6.4)	(15.6)	(8.6)	(8.5)

Source: Authors using NSSO 71^st^ Round on Social Consumption: Health (2014)

Standard error is reported in parenthesis

We also examined the nature of ailment for which different systems of treatment were used (Table 6). In rural areas, ISM had been mostly used for musculo-skeletal, ear and gastro-intestinal ailments and in urban areas it is mostly sought for skin, musculo-skeletal, injuries, and genito-urinary ailments. Homeopathy is sought majorly for skin-related ailments in rural India and for ear and skin-related ailments in urban part. Utilization of Yoga, Naturopathy and ‘others’ is low; accounting for less than 1% in both rural and urban setting. About 4.2% ailing persons in rural and 2.7% in urban sector did not seek any treatment for their ailment.

**Table 6:** Distribution of spells of ailment by nature of ailment and treatment used in last 15 days (excluding hospitalization), Rural and Urban India, 2014.

Nature of ailment	Allopathy	ISM	Homeopathy	Yoga, Naturopathy	Others	No treatment
**Rural India**						
Infection	93.8	1.6	1.7	0.1	0.5	2.3
Cancers	92.4	0.0	0.6	0.0	0.0	7.0
Blood diseases	87.4	3.6	0.0	0.0	0.0	9.0
Endocrine, Metabolic, Nutritional	96.1	1.1	2.3	0.0	0.0	0.5
Psychiatric and Neurological	84.5	4.4	2.0	0.5	0.6	8.0
Eye	87.9	0.4	1.7	0.0	0.9	9.1
Ear	74.5	6.0	3.7	5.6	0.9	9.3
Cardiovascular	96.7	1.0	0.8	0.3	0.1	1.1
Respiratory	85.3	4.2	2.8	0.3	0.1	7.4
Gastro-Intestinal	86.6	4.7	3.8	0.6	0.1	4.3
Skin	79.2	3.4	11.7	0.1	0.5	5.2
Musculo-Skeletal	80.3	7.5	4.1	1.0	0.8	6.3
Genito-Urinary	88.5	1.8	4.0	0.1	0.7	4.8
Obstetric	97.9	0.5	0.4	0.0	1.4	0.0
Injuries	82.3	4.0	0.1	5.2	0.0	8.4
Others, undiagnosed	80.6	2.0	9.1	1.5	0.2	6.5
**All ailments**	**89.2**	**3.1**	**2.7**	**0.5**	**0.4**	**4.2**
**Urban India**						
Infection	94.3	1.5	2.6	0.5	0.2	1.0
Cancers	83.4	1.1	8.8	0.0	0.0	6.7
Blood diseases	84.8	0.7	2.0	0.0	0.4	12.2
Endocrine, Metabolic, Nutritional	94.6	2.2	1.2	0.1	0.2	1.8
Psychiatric and Neurological	88.9	1.8	3.9	0.0	0.3	5.1
Eye	90.3	2.5	1.7	0.0	0.0	5.5
Ear	70.9	4.3	16.9	0.0	0.0	7.9
Cardiovascular	96.6	1.0	1.6	0.0	0.1	0.7
Respiratory	89.1	3.5	3.3	0.1	0.2	3.8
Gastro-Intestinal	90.9	4.5	2.5	0.0	0.0	2.1
Skin	75.3	10.0	10.1	0.0	1.0	3.6
Musculo-Skeletal	76.8	10.0	4.8	0.6	1.0	6.7
Genito-Urinary	86.2	7.3	3.8	0.2	0.2	2.3
Obstetric	95.5	0.0	4.5	0.0	0.0	0.0
Injuries	83.6	8.1	6.1	0.0	2.2	0.1
Others, undiagnosed	76.6	9.0	5.3	0.0	1.0	8.1
**All ailments**	**90.5**	**3.4**	**2.9**	**0.2**	**0.3**	**2.7**

Source: Authors using NSSO 71^st^ Round on Social Consumption: Health (2014)

**Fig 2:**
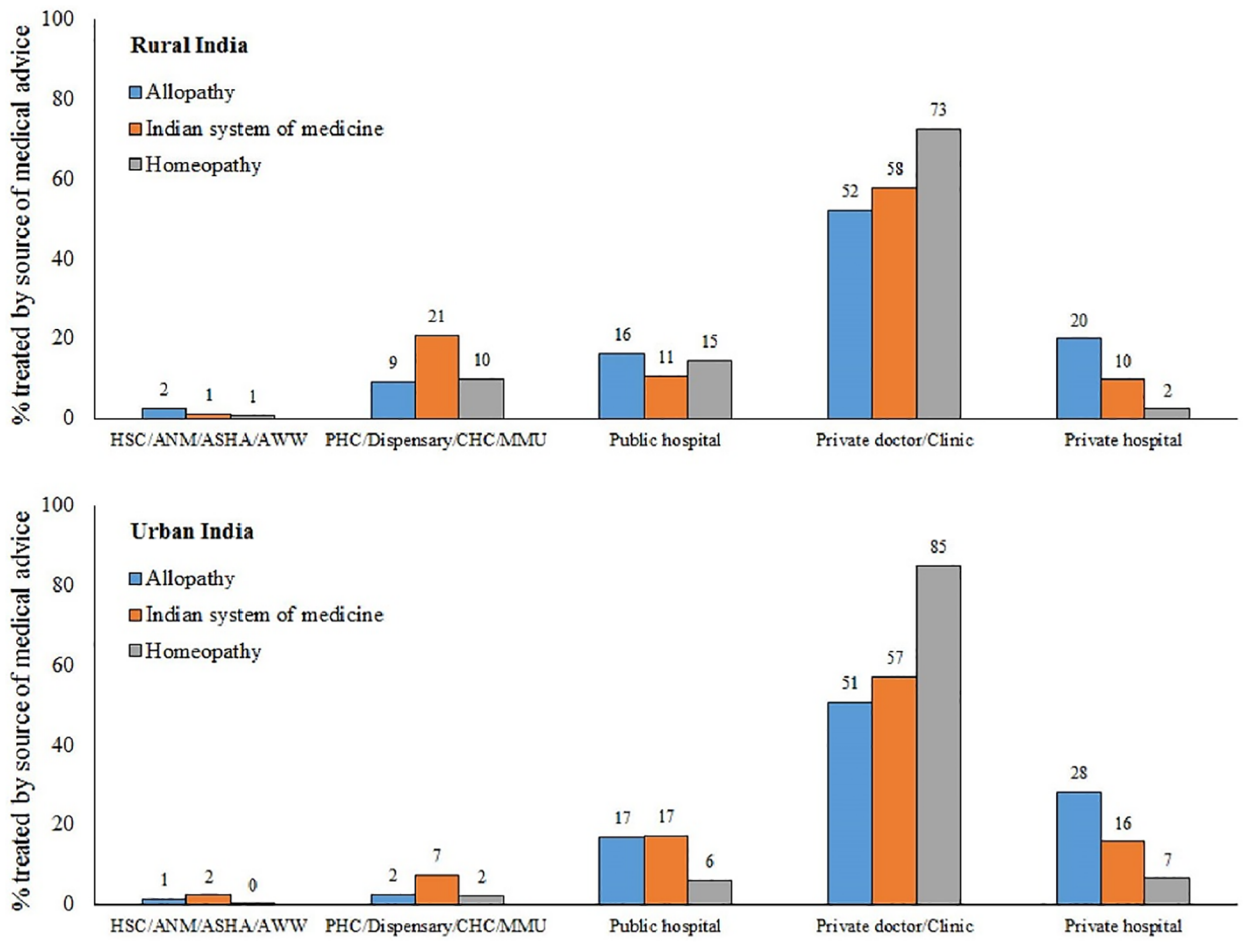
Distribution of source of medical advice by nature of treatment used in last 15 days (excluding hospitalization), Rural and Urban India, 2014. Source: Authors using NSSO 71^st^ Round on Social Consumption: Health (2014).

Fig 2 describes the distribution of sources of medical advice while using different forms of treatment (allopathy, ISM and homeopathy) during outpatient care. These sources of medical advice are categorized into private hospital, public hospitals, private doctor/clinic, health centers and dispensary and community health workers such as auxiliary nurse and midwife at Health Sub Centres (ANM/HSC), accredited social health activist (ASHA), anganwadi worker (AWW). It is no surprise that, private sector providers are the most common source for treatment in India but some variations are worth reporting. In both rural and urban India, about one-half of the treated spells on allopathic medical advice use services of private doctors or clinics. Private hospitals are the second largest allopathy care provider and had a share of 20% and 28% in rural and urban India, respectively. Overall, for allopathy-based outpatient care, public sector had a share of about 25% and 20% in rural and urban parts, respectively. Interestingly, in case of ISM-based outpatient care the treatment share of public sector providers is relatively higher than the allopathy care at 33% and 26% for rural and urban India, respectively. While private doctors and clinics maintained their higher share as care providers, private hospitals had much lower share in ISM-based care. Homeopathy-based outpatient care is largely provided by private doctors or clinics with limited role of public sector.

Table 7 presents the results from two separate probit selection models to understand the correlates associated with use of AYUSH care (Model 1) and the factors influencing use of medical advice for AYUSH care (Model 2). The selection equation from Model 1 shows that in general utilization of outpatient care is significantly higher among females, elderly and those from urban areas. Also, the probability of seeking care increased with economic status of the patients. After adjusting for such probable selection bias, it is observed that use of AYUSH care is higher among females and among children below age 5 years and elderly persons aged 60 and above. Use of AYUSH care is greater among educated individuals but no significant differential is observed across rural and urban areas. Patients from scheduled tribe population are more likely to utilize AYUSH care compared to other social groups. Although, there is no specific income gradient in use of AYUSH care but individuals in the fourth MPCE quintile are relatively less likely to use AYUSH. This finding also corresponds with the observed shape of the concentration curve for AYUSH care. We also run separate logistic regression models (both adjusted and adjusted for primary sampling unit and state level random effects) to test the sensitivity of this result and arrive at similar conclusions (S7 Table). It is also noted that use of AYUSH-based outpatient care also varies across nature of ailment with highest probability of use in case of skin diseases, musculo-skeletal ailments and cancer.

**Table 7:** Probit selection model estimates for correlates of AYUSH use in last 15 days and AYUSH use on medical advice in last 15 days, NSS 2014.

	Model 1 Equations				Model 2 Equations			
	Treatment		Selection		Treatment		Selection	
Dependent variables	Use of AYUSH in last 15 days		Received care in last 15 days		Used AYUSH on medical advice		Use of AYUSH in last 15 days	
Correlates/Variables	coef	se	coef	se	coef	se	coef	se
Rural^®^								
Urban	0.02	0.03	0.16***	0.02	0.26***	0.04	-0.05**	0.02
Male^®^								
Female	0.07***	0.02	0.10***	0.02	-0.04	0.04	0.08***	0.02
Aged 15–59 years ^®^								
Aged 0–5 years	0.14***	0.05	-0.01	0.04	0.10	0.07	0.12***	0.04
Aged 6–14 years	0.01	0.05	-0.27***	0.04	-0.09	0.08	0.04	0.05
Aged 60 years and above	0.08***	0.03	0.44***	0.03	0.04	0.05	-0.02	0.03
Illiterate^®^								
Up to primary education	0.15***	0.03	0.20***	0.03	-0.02	0.06	0.09***	0.03
Up to secondary education	0.16***	0.04	0.16***	0.03	0.05	0.07	0.11***	0.04
Higher education	0.24***	0.05	0.11***	0.04	-0.07	0.08	0.18***	0.04
Scheduled tribe^®^								
Scheduled caste	-0.15***	0.05	0.52***	0.04	0.41***	0.08	-0.19***	0.05
Other backward classes	-0.16***	0.05	0.51***	0.04	0.46***	0.07	-0.21***	0.05
Other social groups	-0.16***	0.05	0.52***	0.04	0.42***	0.07	-0.21***	0.05
Hinduism^®^								
Islam	0.09***	0.03	0.19***	0.03	0.07	0.06	0.08**	0.03
Other religion	-0.03	0.04	0.23***	0.04	0.01	0.07	-0.04	0.04
Poorest MPCE quintile^®^								
Second MPCE quintile	-0.1*	0.04	-0.02	0.04	0.09	0.07	-0.09**	0.04
Third MPCE quintile	-0.03	0.04	0.08**	0.03	0.20***	0.07	-0.08**	0.04
Fourth MPCE quintile	-0.12***	0.04	0.11***	0.03	0.31***	0.07	-0.18***	0.04
Highest MPCE quintile	-0.01	0.04	0.35***	0.03	0.26***	0.06	-0.12***	0.04
Other undiagnosed ailment^®^								
Cancer	1.11***	0.30	4.18***	0.22				
Blood diseases	0.61**	0.28	4.30***	0.14				
Infections	0.61**	0.24	4.45***	0.05				
Endocrine, metabolic, nutrition	0.45*	0.24	5.02***	0.06				
Psychiatric, neurological	0.73***	0.24	4.14***	0.05				
Eye or ear problems	0.67***	0.25	3.90***	0.07				
Cardiovascular diseases	0.34	0.24	5.00***	0.06				
Respiratory diseases	0.85***	0.23	4.06***	0.04				
Gastro-intestinal diseases	0.99***	0.24	4.59***	0.07				
Skin related	1.3***	0.24	4.33***	0.09				
Musculo-skeletal	1.29***	0.23	4.14***	0.04				
Genito-urinary	1.14***	0.25	4.60***	0.12				
Obstetric	0.79**	0.31	8.99	506.1				
Injuries	1.02***	0.25	4.32***	0.14				
Other illness (last 15 days)	-		0.92***	0.03	-		-	
Chronic illness	-		-		-		0.22***	0.03
Constant	-2.40***	0.25	-3.85***	0.05	1.58***	0.08	-1.52***	0.06
Rho	0.43***	0.10			-0.98***	0.02		
Observations	336470				30035			
Censored	306435				28290			
Wald test of Indep. Eqns. (rho = 0)	18.09***				45.26***			
LR Test for instrument	824.89***				82.16***			

Note:

*** p<0.01,

** p<0.05,

* p<0.1;

^®^ denotes the reference category for the particular variable

Treatment without medical advice or self-medication is an important issue in AYUSH care. For instance, in rural and urban India, respectively, about 13% and 8% allopathic medicine based treated spells were not based on direct medical advice. But in case of ISM a greater proportion of treated spells were without direct medical advice (50% and 22% in rural and urban areas, respectively) though use of homeopathy medicine was largely based on medical advice. To understand this behavior, we employ the probit selection model and explore the correlates that may influence treatment based on medical advice. In this regard, the selection equation shows that among individuals receiving treatment, females and children below age 5 are more likely to use AYUSH. The treatment equation in Model 2 shows that patients from urban areas are significantly more likely to use AYUSH based on medical advice. However, no significant differences are observed across age group or by education categories though patients from scheduled tribe population were less likely to use AYUSH based on medical advice. It is also evident that richer households are more likely to use medical advice for AYUSH care (S8 Table). Results from both the models satisfy the robustness test for independence of the two equations as well as for validity of the exclusion restrictions.

## Discussion and conclusion

There is an increasing demand to mainstream AYUSH in India particularly to enhance complementarity and to optimize roles of providers within the formal health care system. However, this will be influenced by factors like availability of infrastructure and human resources, socioeconomic conditions, treatment costs, morbidity patterns and political economy of healthcare services. In this context, understanding the overall level and socioeconomic patterns in utilization of AYUSH care can provide vital insights to proceed further with this important concern. This analysis reveals that about 6.9% of all patients seeking outpatient care (with reference period of last 15 days) had used AYUSH services (3.5% ISM and 3.0% homeopathy). This is consistent with the fact that use of allopathy treatment is more common and that there is hardly any differentials in use pattern across rural and urban India. Also, allopathy care accounted for over 90% of outpatient care across key socioeconomic and demographic variables. Higher utilization of AYUSH care is observed in Chhattisgarh, Kerala and West Bengal even though these states share very distinct profiles. In fact, despite well-developed allopathy based (public and private) systems, ayurveda industry has been growing at a faster pace in Kerala [37] thus suggesting that the industry could be driven by need-based demand. States from North-East India particularly Arunachal Pradesh, Mizoram and Meghalaya also reveal high AYUSH care utilization.

Nevertheless, overall AYUSH utilization in India (about 7% of outpatient care) appears to be on the lower side when compared to some of the previous estimates or general perceptions [8]. For instance, out of the total 18 states surveyed by Priya and Shweta [5], in about five states AYUSH services were reportedly utilized by over 60% households, in another six states about 30–60% households reported utilization and in another five states it is less than 30% households. Similarly, based on a survey of 35 districts across 19 states, Singh et al [29] found that about 14% patients were actually availing ISM and homeopathy treatment whereas analysis of WHO-SAGE survey revealed that 11.7% respondents use traditional medicine as a frequent source of care [30]. Apparently, these estimates are not directly comparable because of the variations in the years of these surveys, use of alternative definitions for traditional medicine and also differences in recall period, reference population and analytical units (household or individual). For instance, the estimates in Priya and Shweta [5] are at the household level and uses a recall period of last 3 months. Similarly, the estimates in Oyebode et al [30] captures only the adult population (18+) and classifies a person as user of traditional medicine if the person has reported at least one consultation with a traditional medicine practitioner in the last 12 months. As desired, the NSS survey captures this information at the individual level and for all the age groups but uses a recall period of last 15 days for outpatient visit. Besides, the NSS survey focuses only on therapeutic use of AYUSH by the ailing individuals and may have ignored use of traditional medicine in disease prevention or health promotion among individuals ailing or otherwise. Given such intricacies and limitations, it will be useful to develop specific guidelines to facilitate consistent spatial and inter-temporal assessment of utilization of various forms of treatment. Although, regular NSS health surveys could be a good source to facilitate such general assessments but a separate and comprehensive survey on AYUSH is more desirable to capture the various aspects associated with AYUSH care. For instance, any such survey should also consider the use of traditional medicine as complementary medicine (along with allopathy treatment). We therefore reiterate that these estimates regarding AYUSH utilization should be viewed in accordance with its purpose and scope to avoid any misleading interpretations including the possibility of labelling key AYUSH policy initiatives as futile exercises [30].

As such, there is limited and mixed evidence to understand the socioeconomic determinants of use of traditional medicine in India. An earlier study by Singh et al [29] found that use of ISM and homeopathy was greater among households with higher income and literacy levels whereas a recent study based on WHO-SAGE survey [30] suggests that those individuals with lower socio-economic status and those living in rural areas were more likely to report use of traditional healers [30]. In this regard, this nationally representative survey presented some interesting conclusions which vary from earlier studies. For instance, it was observed that conditional on receipt of outpatient care when ill, patients with higher educational status are more likely to use AYUSH services. It also emerged that AYUSH use is relatively low among patients in the middle MPCE quintiles. To some extent, this is similar to the Chinese and Nepalese experience where traditional medicine use is more among higher income households [38,39]. In fact, after adjusting for socioeconomic and demographic variables, we did not observe any significant rural-urban differentials in use of AYUSH services for outpatient care though urban households were more likely to seek medical advice for such care. In India, religious beliefs and practices are perceived to be associated with use of different forms of traditional medicine. In this regard, the econometric analysis (Table 7) does suggest that Muslims are more likely to use AYUSH care but due to limited sample limitations we could not undertake separate analysis to discern the association between religious background and use of various forms of traditional medicine. Similarly, it is also true that use of AYUSH care is higher among tribal households but the results show that such households are also less likely to seek medical advice. This perhaps indicates that, among other reasons, poor access to allopathy treatment and probably better knowledge and practice of traditional medicine can be the determining factors. In fact, a study from Nepal also observes that knowledge of medicinal plants can influence greater (self-medication) utilization of traditional medicine [38]. Nevertheless, further research is required to understand nature of self-medication and knowledge and awareness levels of such households.

Like most developing countries, Indian healthcare system is characterized by low health budget, poor service delivery, high absenteeism and a rigid rural-urban dichotomy. The publicly-funded rural healthcare network of sub-centres (SC), primary health centres (PHC) and community health centres (CHC) are grossly deficit as per norms laid down by the WHO. Amidst all of this, there is a burgeoning unregulated private healthcare market in cities and villages. Therefore, it is no surprise to observe a dominant presence of private sector in providing both allopathy and AYUSH outpatient care services. In fact, the private sector accounts for about three-fourth of the total outpatient care visits. Within the private sector, a major share of health care provision was scattered across private doctors or clinics. In this context, it is important to note that in rural areas provisioning of AYUSH services for outpatient care was significantly driven by public health facilities. In fact, about one-fifth of total ISM-based outpatient care was provided at PHCs or CHCs. This hints at the important role that National Rural Health Mission (NRHM) has played in promoting AYUSH care by placing AYUSH practitioners in public health facilities across rural areas. This is corroborated by Priya and Shweta [5] who reported fairly good attendance for outpatient care across public health facilities providing AYUSH care including those co-located in the public health system under NRHM.

Many studies have unequivocally highlighted the massive presence of informal practitioners of alternate medicine in rural and urban India [2,7,8, 21,22,40]. Although, the NSS survey does not facilitate an analysis regarding eligibility and qualifications of health care providers but this structure of health care provision certainly has implications for out of pocket expenditure. While the overall share of AYUSH medicine in total medicine expenditure was only about 6% but the average AYUSH medicine expenditure per AYUSH treated person (Rs. 270 in rural and Rs. 378 in urban) did not hugely differ from average allopathy medicine expenditures (Rs. 392 in rural and Rs. 454 in urban). However, since reasonable proportions of AYUSH care is now sourced from public sector therefore, it is possible that improved provisioning of free AYUSH medicines can reduce the average out of pocket medicinal expenditure. In this context, policies have to also focus on the dichotomy between rural-urban and public-private sector. While subsidized public healthcare in cities largely caters to the relatively better-off sections of the society, the rural healthcare system is either understaffed or manned by functionaries who are not equipped to give clinical care thus leading to high dependency on private providers with varying skills and competence [41,42]. In fact, better quality private healthcare services are iniquitous because they are profit-making (thus making them financially inaccessible) and urban centric [43]. This has also allowed the informal healthcare providers to occupy the vacuum created by dysfunctional public health system and inaccessible private healthcare services [44–47].

From a policy perspective, it is also useful to note that use of AYUSH services is significantly higher for treating skin-related or musculo-skeletal related ailments [48]. The utilization of AYUSH for acute illnesses is lower than for chronic diseases in both rural and urban areas, reflecting an inclination of the users for AYUSH often when treatment is long term. This pattern suggests possibilities of expanding the role of AYUSH care, particularly for managing chronic diseases. For instance, McDowell and Pai [49] have illustrated the role of AYUSH providers in TB control in Mumbai which provides insights for further scope for integration. Some studies have also argued for expanding the role of public sector in provisioning of AYUSH care for maternity services or gynaecological disorders by effectively linking allopathic and non-allopathic medicine and the concerned human resources including traditional birth attendants and AYUSH providers [50,51]. But any such move towards medical pluralism is not devoid of potential conflicts related to theoretical ideologies, medical practice, resource allocations, professional status, effectiveness and ethics [8,9, 22,52,53]. Clearly, as argued by Lakshmi et al [22] and Powell-Jackson [49], achieving co-operation among different systems and delivery of quality care services, would be enormous challenges. Although, at present there is some encouraging evidence [49,54,55] to establish the effectiveness and impact of integrated medicine but more empirical support is warranted. In this regard, a gradual yet progressive approach would be to identify domains where integration may have little or no scope for conflicts. Besides, training and drawing up effective regulations for rural health practitioners [56] or for promoting traditional medicine, particularly in rural areas, is a long-pending demand and on this issue there are important lessons to be learnt from other Asian countries [4,21]. In fact, the health system in China has successfully augmented human resources for traditional medicine and effectively integrated conventional medicine at every tier of health-care and has also made provisions for public and private insurance cover for both traditional Chinese medicine and conventional medicine [2,57].

To round up the discussion, it is worth highlighting the need for accreditation and certification of traditional healing practices requiring extensive codification of folk practices. Also, efforts and strategies are needed for sustaining and expanding the knowledge base of traditional medicine for effective use. For instance, the NSSO 68^th^ round data on household consumer expenditure survey reveals that lack of awareness was one of the major reasons cited for not using AYUSH care (S1 Fig). Similarly, another important area would be to address concerns related to perceived non-effectiveness of AYUSH care (S9 and S10 Tables) [58]. This calls for greater discussion regarding scientific merits, health benefits and cost-effectiveness of AYUSH medicine. For instance, the property of low or no side-effects is often considered as a merit of traditional medicine that drives its acceptance in the community [55,59,60] and could be prioritized under preventive care. Moreover, pluralistic form of medicine is the new emerging alternative or parallel to the existing modern medicine (allopathic). In this regard, it is encouraging that Government of India has been very progressive in institutionalizing AYUSH health care services but in this regard a national policy for mainstreaming AYUSH is highly desirable.

## Supporting information

S1 Fig Concentration curves for nature of treatment–Allopathy and ISM—In last 15 days (excluding hospitalization), Rural and Urban India, 2014. Source: Authors using NSSO 71st Round on Social Consumption: Health (2014).
(TIF)

S2 Fig Distribution of source of medical advice by nature of treatment used in last 15 days (excluding hospitalization), Rural and Urban India, 2014. Source: Authors using NSSO 71st Round on Social Consumption: Health (2014).
(TIF)

S1 Table Percentage patients (persons reporting illness during reference period of last 15 days) receiving medical treatment (excluding hospitalization) by nature of treatment and background characteristics, Rural India, 2014 (n = 13927). Source: Authors using NSSO 71st Round on Social Consumption: Health (2014). It may be noted that some individuals may have received treatment from more than one forms of medicine and therefore the distribution of patients under nature of treatment is not mutually exclusive.
(DOCX)

S2 Table Percentage patients (persons reporting illness during reference period of last 15 days) receiving medical treatment (excluding hospitalization) by nature of treatment and background characteristics, Urban India, 2014 (n = 13505). Source: Authors using NSSO 71st Round on Social Consumption: Health (2014). It may be noted that some individuals may have received treatment from more than one forms of medicine and therefore the distribution of patients under nature of treatment is not mutually exclusive.
(DOCX)

S3 Table Percentage patients (persons reporting illness during reference period of last 15 days) receiving medical treatment (excluding hospitalization) by nature of treatment and State/UT, 2014. Source: Authors using NSSO 71st Round on Social Consumption: Health (2014). It may be noted that some individuals may have received treatment from more than one forms of medicine and therefore the distribution of patients under nature of treatment is not mutually exclusive.
(DOCX)

S4 Table Concentration indices for nature of treatment used in last 15 days (excluding hospitalization), Rural and Urban India, 2014. Source: Authors using NSSO 71st Round on Social Consumption: Health (2014). Note: Standard error of the CI in parenthesis. * Denotes significance at 5% level.
(DOCX)

S5 Table Average out of pocket expenditure on AYUSH medicines and other (non-AYUSH) medicines per treated person in the last 15 days by sex and place of residence, 2014. Source: Authors using NSSO 71st Round on Social Consumption: Health (2014). Standard error is reported in parenthesis.
(DOCX)

S6 Table Distribution of spells of ailment by nature of ailment and treatment used in last 15 days (excluding hospitalization), Rural and Urban India, 2014. Source: Authors using NSSO 71st Round on Social Consumption: Health (2014).
(DOCX)

S7 Table Probit selection model estimates for correlates of AYUSH use in last 15 days and AYUSH use on medical advice in last 15 days, NSS 2014. Note: *** p<0.01, ** p<0.05, * p<0.1;^®^ denotes the reference category for the particular variable.
(DOCX)
