# Supplementary figures and images for: Utilization of alternative systems of medicine as health care services in India: Evidence on AYUSH care from NSS 2014
Source: PLoS One. 2017 May 4;12(5):e0176916. doi: 10.1371/journal.pone.0176916 (PMC5417584; doi:10.1371/journal.pone.0176916)

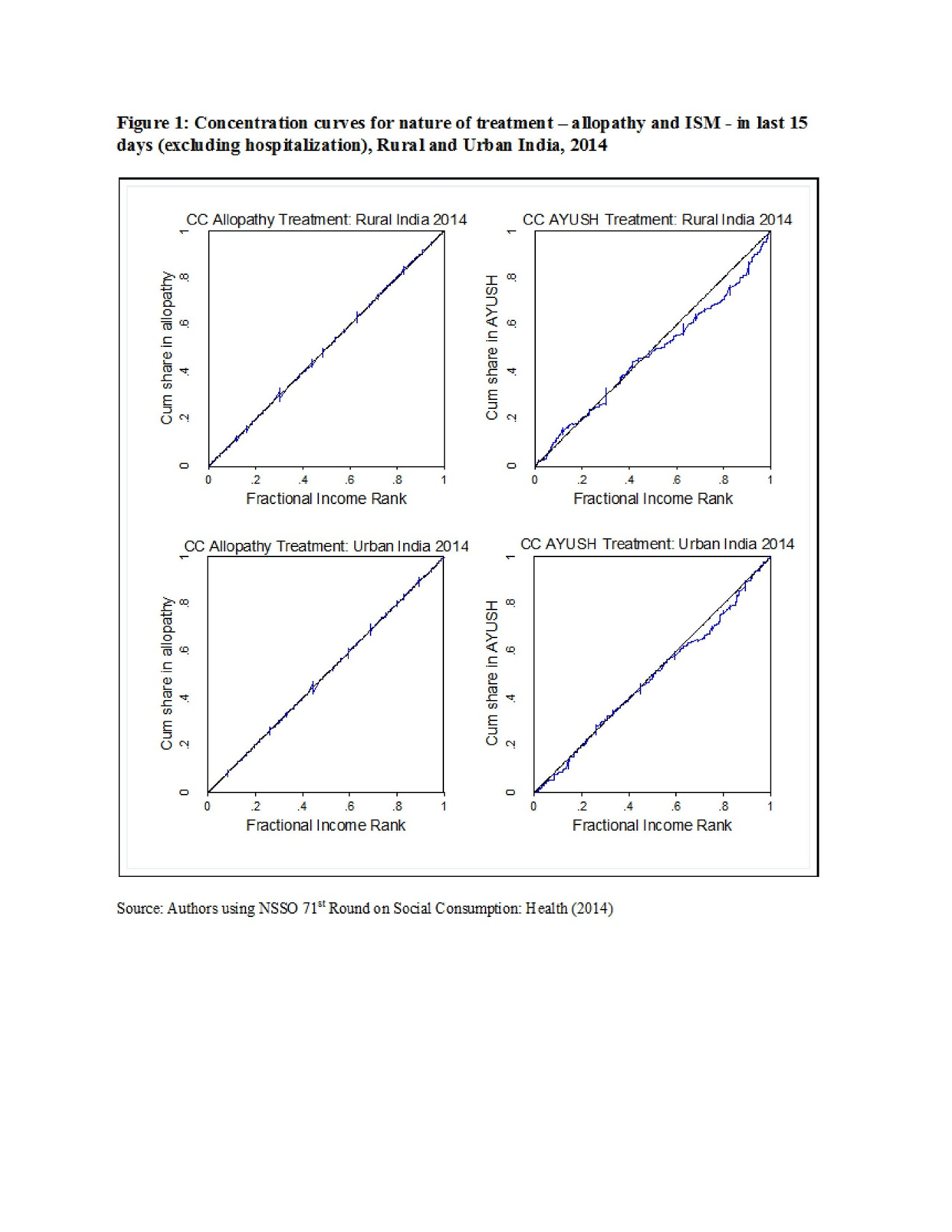

Supplement: S1 Fig — Source: Authors using NSSO 71st Round on Social Consumption: Health (2014). (TIF) [file pone.0176916.s001.tif]

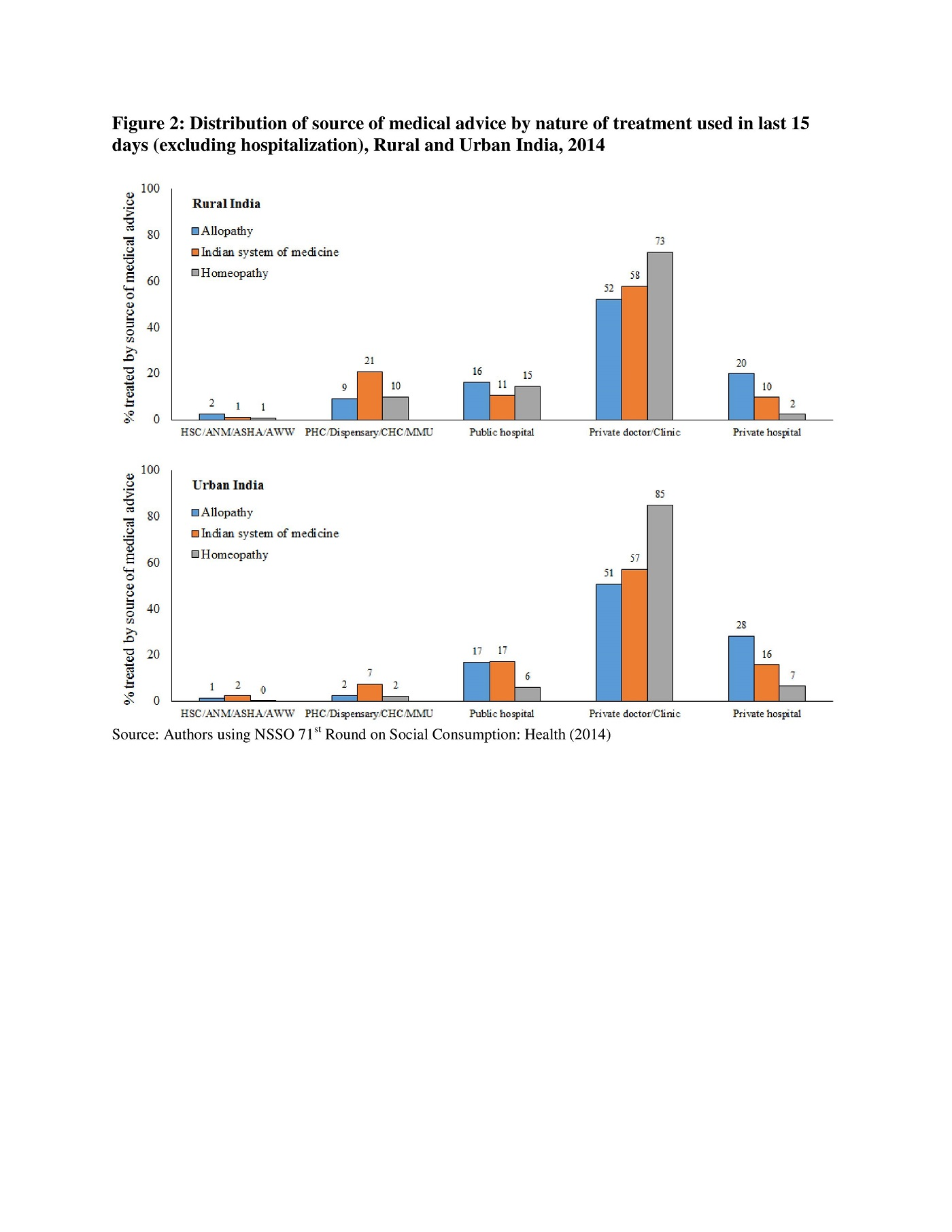

Supplement: S2 Fig — Source: Authors using NSSO 71st Round on Social Consumption: Health (2014). (TIF) [file pone.0176916.s002.tif]
