# Supplementary material for: Utilization of alternative systems of medicine as health care services in India: Evidence on AYUSH care from NSS 2014
Source: PLoS One. 2017 May 4;12(5):e0176916. doi: 10.1371/journal.pone.0176916 (PMC5417584; doi:10.1371/journal.pone.0176916)
Supplement: S2 Table — Source: Authors using NSSO 71st Round on Social Consumption: Health (2014). It may be noted that some individuals may have received treatment from more than one forms of medicine and therefore the distribution of patients under nature of treatment is not mutually exclusive. (DOCX) [file pone.0176916.s004.docx]

**Table 2: Percentage patients (persons reporting illness during reference period of last 15 days) receiving medical treatment (excluding hospitalization) by nature of treatment and background characteristics, Urban India, 2014 (n=13505)**

| **Urban India** | **Allopathy** | **ISM** | **Homoeopathy** | **Yoga & Naturopathy** | **Other** | **AYUSH** |
| --- | --- | --- | --- | --- | --- | --- |
| **Age** |  |  |  |  |  |  |
| Below 5 years | 92.2 | 2.9 | 4.3 | 0.3 | 0.4 | 7.4 |
| 5 to 14 years | 93.8 | 1.6 | 3.8 | 0.9 | 0.0 | 6.3 |
| 15 to 59 years | 93.5 | 3.8 | 3.2 | 0.2 | 0.5 | 7.2 |
| 60 years and above | 93.8 | 4.8 | 2.4 | 0.1 | 0.1 | 7.2 |
| **Sex** |  |  |  |  |  |  |
| Male | 93.2 | 4.4 | 2.4 | 0.3 | 0.5 | 7.1 |
| Female | 93.8 | 3.2 | 3.7 | 0.2 | 0.2 | 7.1 |
| **Social group** |  |  |  |  |  |  |
| Scheduled Tribes | 94.5 | 3.5 | 1.7 | 0.6 | 0.0 | 5.8 |
| Scheduled Castes | 94.5 | 4.1 | 2.0 | 0.2 | 0.2 | 6.3 |
| Other Backward Classes | 93.6 | 4.0 | 3.0 | 0.2 | 0.3 | 7.3 |
| Others | 93.0 | 3.3 | 3.7 | 0.3 | 0.5 | 7.3 |
| **Religion** |  |  |  |  |  |  |
| Hinduism | 93.5 | 3.8 | 3.1 | 0.3 | 0.3 | 7.2 |
| Islam | 93.2 | 3.2 | 3.9 | 0.0 | 0.5 | 7.1 |
| Others | 94.2 | 4.1 | 2.2 | 0.1 | 0.1 | 6.4 |
| **Education of Head** |  |  |  |  |  |  |
| Illiterate | 94.3 | 2.9 | 3.0 | 0.1 | 0.2 | 5.9 |
| Primary or below | 94.1 | 3.3 | 3.5 | 0.2 | 0.2 | 7.0 |
| Secondary education | 93.6 | 4.0 | 2.4 | 0.6 | 0.3 | 6.9 |
| Higher education | 91.6 | 5.1 | 3.9 | 0.0 | 0.8 | 9.0 |
| **MPCE quintile** |  |  |  |  |  |  |
| Lowest | 94.9 | 2.3 | 2.9 | 0.1 | 0.1 | 5.3 |
| Second | 91.8 | 4.8 | 3.8 | 0.1 | 0.3 | 8.7 |
| Middle | 93.6 | 3.1 | 3.1 | 0.8 | 0.1 | 7.0 |
| Fourth | 94.9 | 3.3 | 1.8 | 0.1 | 0.7 | 5.2 |
| Highest | 92.7 | 4.6 | 4.0 | 0.1 | 0.4 | 8.7 |
| **Chronic illness** |  |  |  |  |  |  |
| Yes | 93.1 | 4.4 | 3.6 | 0.2 | 0.5 | 8.1 |
| **Acute illness** |  |  |  |  |  |  |
| Yes | 94.1 | 2.9 | 3.0 | 0.3 | 0.3 | 6.2 |
| **Urban India** | 93.5 | 3.7 | 3.2 | 0.2 | 0.3 | 7.1 |

Source: Authors using NSSO 71^st^ Round on Social Consumption: Health (2014)

It may be noted that some individuals may have received treatment from more than one forms of medicine and therefore the distribution of patients under nature of treatment is not mutually exclusive.
