# Supplementary material for: Utilization of alternative systems of medicine as health care services in India: Evidence on AYUSH care from NSS 2014
Source: PLoS One. 2017 May 4;12(5):e0176916. doi: 10.1371/journal.pone.0176916 (PMC5417584; doi:10.1371/journal.pone.0176916)
Supplement: S4 Table — Source: Authors using NSSO 71st Round on Social Consumption: Health (2014). Note: Standard error of the CI in parenthesis. * Denotes significance at 5% level. (DOCX) [file pone.0176916.s006.docx]

**Table 4: Concentration indices for nature of treatment used in last 15 days (excluding hospitalization), Rural and Urban India, 2014**

| **Nature of treatment** | **CI for Rural India (se)** | | **CI for Urban India (se)** | |
| --- | --- | --- | --- | --- |
| Allopathy | -0.001 | *(0.001)* | -0.001 | *(0.001)* |
| Indian System of Medicine | 0.064* | *(0.026)* | 0.074* | *(0.025)* |
| Homeopathy | 0.049 | *(0.028)* | -0.008 | *(0.028)* |
| Yoga and Naturopathy | -0.006 | *(0.068)* | -0.177 | *(0.102)* |
| Other treatment | -0.062 | *(0.078)* | 0.239* | *(0.085)* |
| AYUSH | 0.047* | *(0.018)* | 0.029 | *(0.018)* |

Source: Authors using NSSO 71^st^ Round on Social Consumption: Health (2014)

Note: *Standard error* of the CI in parenthesis

* Denotes significance at 5% level.
