# Supplementary material for: Utilization of alternative systems of medicine as health care services in India: Evidence on AYUSH care from NSS 2014
Source: PLoS One. 2017 May 4;12(5):e0176916. doi: 10.1371/journal.pone.0176916 (PMC5417584; doi:10.1371/journal.pone.0176916)
Supplement: S5 Table — Source: Authors using NSSO 71st Round on Social Consumption: Health (2014). Standard error is reported in parenthesis. (DOCX) [file pone.0176916.s007.docx]

**Table 5: Average out of pocket expenditure on AYUSH medicines and other (non-AYUSH) medicines per treated person in the last 15 days by sex and place of residence, 2014**

| **OOP expenditure (in Rupee)** | **Rural India (std. err)** | | | **Urban India (std. err)** | | |
| --- | --- | --- | --- | --- | --- | --- |
|  | **Male** | **Female** | **All** | **Male** | **Female** | **All** |
| AYUSH medicine | 322 | 228 | 270 | 462 | 311 | 378 |
|  | (37.4) | (16.0) | (19.2) | (36.9) | (26.4) | (22.1) |
| Other (non-AYUSH) medicine | 381 | 402 | 392 | 485 | 430 | 454 |
|  | (7.8) | (10.0) | (6.4) | (15.6) | (8.6) | (8.5) |

Source: Authors using NSSO 71^st^ Round on Social Consumption: Health (2014)

Standard error is reported in parenthesis
