# Supplementary material for: Utilization of alternative systems of medicine as health care services in India: Evidence on AYUSH care from NSS 2014
Source: PLoS One. 2017 May 4;12(5):e0176916. doi: 10.1371/journal.pone.0176916 (PMC5417584; doi:10.1371/journal.pone.0176916)
Supplement: S6 Table — Source: Authors using NSSO 71st Round on Social Consumption: Health (2014). (DOCX) [file pone.0176916.s008.docx]

**Table 6: Distribution of spells of ailment by nature of ailment and treatment used in last 15 days (excluding hospitalization), Rural and Urban India, 2014**

| **Nature of ailment** | **Allopathy** | **ISM** | **Homeopathy** | **Yoga, Naturopathy** | **Others** | **No treatment** |
| --- | --- | --- | --- | --- | --- | --- |
| **Rural India** |  |  |  |  |  |  |
| Infection | 93.8 | 1.6 | 1.7 | 0.1 | 0.5 | 2.3 |
| Cancers | 92.4 | 0.0 | 0.6 | 0.0 | 0.0 | 7.0 |
| Blood diseases | 87.4 | 3.6 | 0.0 | 0.0 | 0.0 | 9.0 |
| Endocrine, Metabolic, Nutritional | 96.1 | 1.1 | 2.3 | 0.0 | 0.0 | 0.5 |
| Psychiatric and Neurological | 84.5 | 4.4 | 2.0 | 0.5 | 0.6 | 8.0 |
| Eye | 87.9 | 0.4 | 1.7 | 0.0 | 0.9 | 9.1 |
| Ear | 74.5 | 6.0 | 3.7 | 5.6 | 0.9 | 9.3 |
| Cardiovascular | 96.7 | 1.0 | 0.8 | 0.3 | 0.1 | 1.1 |
| Respiratory | 85.3 | 4.2 | 2.8 | 0.3 | 0.1 | 7.4 |
| Gastro-Intestinal | 86.6 | 4.7 | 3.8 | 0.6 | 0.1 | 4.3 |
| Skin | 79.2 | 3.4 | 11.7 | 0.1 | 0.5 | 5.2 |
| Musculo-Skeletal | 80.3 | 7.5 | 4.1 | 1.0 | 0.8 | 6.3 |
| Genito-Urinary | 88.5 | 1.8 | 4.0 | 0.1 | 0.7 | 4.8 |
| Obstetric | 97.9 | 0.5 | 0.4 | 0.0 | 1.4 | 0.0 |
| Injuries | 82.3 | 4.0 | 0.1 | 5.2 | 0.0 | 8.4 |
| Others, undiagnosed | 80.6 | 2.0 | 9.1 | 1.5 | 0.2 | 6.5 |
| **All ailments** | **89.2** | **3.1** | **2.7** | **0.5** | **0.4** | **4.2** |
| **Urban India** |  |  |  |  |  |  |
| Infection | 94.3 | 1.5 | 2.6 | 0.5 | 0.2 | 1.0 |
| Cancers | 83.4 | 1.1 | 8.8 | 0.0 | 0.0 | 6.7 |
| Blood diseases | 84.8 | 0.7 | 2.0 | 0.0 | 0.4 | 12.2 |
| Endocrine, Metabolic, Nutritional | 94.6 | 2.2 | 1.2 | 0.1 | 0.2 | 1.8 |
| Psychiatric and Neurological | 88.9 | 1.8 | 3.9 | 0.0 | 0.3 | 5.1 |
| Eye | 90.3 | 2.5 | 1.7 | 0.0 | 0.0 | 5.5 |
| Ear | 70.9 | 4.3 | 16.9 | 0.0 | 0.0 | 7.9 |
| Cardiovascular | 96.6 | 1.0 | 1.6 | 0.0 | 0.1 | 0.7 |
| Respiratory | 89.1 | 3.5 | 3.3 | 0.1 | 0.2 | 3.8 |
| Gastro-Intestinal | 90.9 | 4.5 | 2.5 | 0.0 | 0.0 | 2.1 |
| Skin | 75.3 | 10.0 | 10.1 | 0.0 | 1.0 | 3.6 |
| Musculo-Skeletal | 76.8 | 10.0 | 4.8 | 0.6 | 1.0 | 6.7 |
| Genito-Urinary | 86.2 | 7.3 | 3.8 | 0.2 | 0.2 | 2.3 |
| Obstetric | 95.5 | 0.0 | 4.5 | 0.0 | 0.0 | 0.0 |
| Injuries | 83.6 | 8.1 | 6.1 | 0.0 | 2.2 | 0.1 |
| Others, undiagnosed | 76.6 | 9.0 | 5.3 | 0.0 | 1.0 | 8.1 |
| **All ailments** | **90.5** | **3.4** | **2.9** | **0.2** | **0.3** | **2.7** |

Source: Authors using NSSO 71^st^ Round on Social Consumption: Health (2014)
