# Supplementary material for: Utilization of alternative systems of medicine as health care services in India: Evidence on AYUSH care from NSS 2014
Source: PLoS One. 2017 May 4;12(5):e0176916. doi: 10.1371/journal.pone.0176916 (PMC5417584; doi:10.1371/journal.pone.0176916)
Supplement: S7 Table — Note: *** p<0.01, ** p<0.05, * p<0.1;® denotes the reference category for the particular variable. (DOCX) [file pone.0176916.s009.docx]

**Table 7: Probit selection model estimates for correlates of AYUSH use in last 15 days and AYUSH use on medical advice in last 15 days, NSS 2014**

|  | **Model 1 Equations** | | | | **Model 2 Equations** | | | |
| --- | --- | --- | --- | --- | --- | --- | --- | --- |
|  | **Treatment** | | **Selection** | | **Treatment** | | **Selection** | |
| Dependent variables | **Use of AYUSH in last 15 days** | | **Received care in last 15 days** | | **Used AYUSH on medical advice** | | **Use of AYUSH in last 15 days** | |
| Correlates/Variables | **coef** | **se** | **coef** | **se** | **coef** | **se** | **coef** | **se** |
| Rural® |  |  |  |  |  |  |  |  |
| Urban | 0.02 | 0.03 | 0.16*** | 0.02 | 0.26*** | 0.04 | -0.05** | 0.02 |
| Male® |  |  |  |  |  |  |  |  |
| Female | 0.07*** | 0.02 | 0.10*** | 0.02 | -0.04 | 0.04 | 0.08*** | 0.02 |
| Aged 15 – 59 years ® |  |  |  |  |  |  |  |  |
| Aged 0-5 years | 0.14*** | 0.05 | -0.01 | 0.04 | 0.10 | 0.07 | 0.12*** | 0.04 |
| Aged 6 -14 years | 0.01 | 0.05 | -0.27*** | 0.04 | -0.09 | 0.08 | 0.04 | 0.05 |
| Aged 60 years and above | 0.08*** | 0.03 | 0.44*** | 0.03 | 0.04 | 0.05 | -0.02 | 0.03 |
| Illiterate® |  |  |  |  |  |  |  |  |
| Up to primary education | 0.15*** | 0.03 | 0.20*** | 0.03 | -0.02 | 0.06 | 0.09*** | 0.03 |
| Up to secondary education | 0.16*** | 0.04 | 0.16*** | 0.03 | 0.05 | 0.07 | 0.11*** | 0.04 |
| Higher education | 0.24*** | 0.05 | 0.11*** | 0.04 | -0.07 | 0.08 | 0.18*** | 0.04 |
| Scheduled tribe® |  |  |  |  |  |  |  |  |
| Scheduled caste | -0.15*** | 0.05 | 0.52*** | 0.04 | 0.41*** | 0.08 | -0.19*** | 0.05 |
| Other backward classes | -0.16*** | 0.05 | 0.51*** | 0.04 | 0.46*** | 0.07 | -0.21*** | 0.05 |
| Other social groups | -0.16*** | 0.05 | 0.52*** | 0.04 | 0.42*** | 0.07 | -0.21*** | 0.05 |
| Hinduism® |  |  |  |  |  |  |  |  |
| Islam | 0.09*** | 0.03 | 0.19*** | 0.03 | 0.07 | 0.06 | 0.08** | 0.03 |
| Other religion | -0.03 | 0.04 | 0.23*** | 0.04 | 0.01 | 0.07 | -0.04 | 0.04 |
| Poorest MPCE quintile® |  |  |  |  |  |  |  |  |
| Second MPCE quintile | -0.1* | 0.04 | -0.02 | 0.04 | 0.09 | 0.07 | -0.09** | 0.04 |
| Third MPCE quintile | -0.03 | 0.04 | 0.08** | 0.03 | 0.20*** | 0.07 | -0.08** | 0.04 |
| Fourth MPCE quintile | -0.12*** | 0.04 | 0.11*** | 0.03 | 0.31*** | 0.07 | -0.18*** | 0.04 |
| Highest MPCE quintile | -0.01 | 0.04 | 0.35*** | 0.03 | 0.26*** | 0.06 | -0.12*** | 0.04 |
| Other undiagnosed ailment® |  |  |  |  |  |  |  |  |
| Cancer | 1.11*** | 0.30 | 4.18*** | 0.22 |  |  |  |  |
| Blood diseases | 0.61** | 0.28 | 4.30*** | 0.14 |  |  |  |  |
| Infections | 0.61** | 0.24 | 4.45*** | 0.05 |  |  |  |  |
| Endocrine, metabolic, nutrition | 0.45* | 0.24 | 5.02*** | 0.06 |  |  |  |  |
| Psychiatric, neurological | 0.73*** | 0.24 | 4.14*** | 0.05 |  |  |  |  |
| Eye or ear problems | 0.67*** | 0.25 | 3.90*** | 0.07 |  |  |  |  |
| Cardiovascular diseases | 0.34 | 0.24 | 5.00*** | 0.06 |  |  |  |  |
| Respiratory diseases | 0.85*** | 0.23 | 4.06*** | 0.04 |  |  |  |  |
| Gastro-intestinal diseases | 0.99*** | 0.24 | 4.59*** | 0.07 |  |  |  |  |
| Skin related | 1.3*** | 0.24 | 4.33*** | 0.09 |  |  |  |  |
| Musculo-skeletal | 1.29*** | 0.23 | 4.14*** | 0.04 |  |  |  |  |
| Genito-urinary | 1.14*** | 0.25 | 4.60*** | 0.12 |  |  |  |  |
| Obstetric | 0.79** | 0.31 | 8.99 | 506.1 |  |  |  |  |
| Injuries | 1.02*** | 0.25 | 4.32*** | 0.14 |  |  |  |  |
| Other illness (last 15 days) | - |  | 0.92*** | 0.03 | - |  | - |  |
| Chronic illness | - |  | - |  | - |  | 0.22*** | 0.03 |
| Constant | -2.40*** | 0.25 | -3.85*** | 0.05 | 1.58*** | 0.08 | -1.52*** | 0.06 |
| Rho | 0.43*** | 0.10 |  |  | -0.98*** | 0.02 |  |  |
| Observations | 336470 |  |  |  | 30035 |  |  |  |
| Censored | 306435 |  |  |  | 28290 |  |  |  |
| Wald test of Indep. Eqns. (rho = 0) | 18.09*** |  |  |  | 45.26*** |  |  |  |
| LR Test for instrument | 824.89*** |  |  |  | 82.16*** |  |  |  |

Note: *** p<0.01, ** p<0.05, * p<0.1; ® denotes the reference category for the particular variable
